# Supplementary material for: Intermittent pneumatic compression therapy as a preventive measure for venous thromboembolism after total hip arthroplasty: A systematic review
Source: PLoS One. 2025 Jun 4;20(6):e0318954. doi: 10.1371/journal.pone.0318954 (PMC12136298; doi:10.1371/journal.pone.0318954)
Supplement: S1 Text — (DOCX) [file pone.0318954.s001.docx]

Studies Included and Excluded

| S.No | Authors Name | Year | Title | Criteria |
| --- | --- | --- | --- | --- |
| 1. | Kwak. S et al | 2016 | Intermittent pneumatic compression for the prevention of venous thromboembolism after total hip arthroplasty. | Included |
| 2. | Paudel. S et al | 2019 | Efficacy and safety of Aspirin plus intermittent pneumatic compression device as thromboprophylaxis after total hip arthroplasty. | Included |
| 3. | Pellino. C et al | 2023 | Pneumatic Compression Combined with Standard Treatment after Total Hip Arthroplasty and Its Effects on Edema of the Operated Limb and on Physical Outcomes. | Included |
| 4. | Wang. D et al., | 2018 | Semiautomatic intermittent pneumatic compression device applied to deep vein thrombosis in major orthopedic surgery. | Included |
| 5. | Colwell jr. W et al., | 2010 | Thrombosis Prevention After Total Hip Arthroplasty: a prospective, randomized trail comparing a mobile compression device with low-molecular-weight heparin. | Included |
| 6. | Liqun. W et al., | 2020 | Effect of different use time of intermittent pneumatic compressiom on the incidence of deep vein thrombosis of lower extermities after arthroplasty. | Included |
| 7. | Silbersack.Y et al | 2004 | Prevention of deep-vein thrombosis after total hip and knee replacement: low-molecular-weight heparin in combination with intermittent pneumatic compression. | Included |
| 8. | Eisele R. et al | 2007 | Rapid-inflation intermittent pneumatic compression for prevention of deep venous thrombosis. | Included |
| 9. | Khalafallah. A et al. | 2018 | Assessment of Post-Operative Bleeding and Venous Thromboembolism after Initial 24-Hour Intermittent Pneumatic Calf Compression Followed by Rivaroxaban versus Enoxaparin in Elective Hip and Knee Arthroplasty. | Included |
| 10. | Paudel. S et al | 2014 | Low Molecular Weight Heparin versus Aspirin plus Intermittent Compression Devices for Thromboprophylaxis in Indian Patients Undergoing Total Hip and Knee Arthroplasty. | Included |
| 11. | Ben-Galim P. et al | 2009 | A miniature and mobile intermittent pneumatic compression device for the prevention of deep- vein thrombosis after joint replacement. | Included |
| 12 | Yokote R. et al | 2011 | Is routine chemical thromboprophylaxis after total hip replacement really necessary in a Japanese population ? | Included |
| 13. | Jo WL. et al, | 2016 | Preventing venous thromboembolism with use of intermittent pneumatic compression after total hip arthroplasty in korean patients | Excluded  Reason: Not RCT |
| 14. | Zhao JM. et al, | 2014 | Different types of intermittent pneumatic compression devices for preventing venous thromboembolism in patients after total hip replacement. | Excluded  Reason: Not RCT |
| 15. | Greet W. et al, | 2004 | Prevention of venous thromboembolism: the Seventh ACCP Conference on Antithrombotic and Thrombolytic Therapy | Excluded  Reason: Not RCT |
| 16. | Kakkos S. et al, | 2012 | Combined intermittent pneumatic leg compression and pharmacological prophylaxis for prevention of venous thromboembolism. | Excluded  Reason: Not RCT |
| 17. | Lieberman JR. et al, | 2013 | Prevention of venous thromboembolic disease after total hip and knee arthroplasty. | Excluded  Reason: Not RCT |
| 18. | Pierce Todd. et al, | 2015 | A Current Review of Mechanical Compression and Its Role in Venous Thromboembolic Prophylaxis in Total Knee and Total Hip Arthroplasty. | Excluded  Reason: Not RCT |
| 19. | Colwell jr. W et al., | 2014 | A mobile compression device for thrombosis prevention in hip and knee arthroplasty. | Excluded  Reason: Not RCT |
| 20. | Beksac B. et al, | 2006 | Thromboembolic disease after total hip arthroplasty: who is at risk? | Excluded  Reason: Not RCT |
| 21. | Torres T Rafael. et al, | 2019 | A comparative cost-effectiveness analysis of mechanical and pharmacological VTE prophylaxis after lower limb arthroplasty in Australia | Excluded  Reason: Not RCT |
| 22. | Elbuluk A. et al, | 2017 | Respiratory Synchronized Versus Intermittent Pneumatic Compression in Prevention of Venous Thromboembolism After Total Joint Arthroplasty: A Systematic Review and Meta-Analysis | Excluded  Reason: Not RCT |
| 23. | Pavon J. et al, | 2015 | Effectiveness of Intermittent Pneumatic Compression Devices for Venous Thromboembolism Prophylaxis in High-Risk Surgical Patients: A Systematic Review | Excluded  Reason: Not RCT |
| 24. | Pellegrini Jr. et al, | 2008 | Venous thromboembolic disease after total hip and knee arthroplasty | Excluded  Reason: Not RCT |
| 25. | Haynes J. et al, | 2016 | Mobile pump deep vein thrombosis prophylaxis: just say no to drugs | Excluded  Reason: Not RCT |
| 26. | Arsoy D. et al, | 2018 | Mobile Compression Reduces Bleeding-related Readmissions and Wound Complications After THA and TKA | Excluded  Reason: Not RCT |
| 27. | Masri B. et al, | 2004 | Can a new design of pneumatic compression device reduce variations in delivered therapy for the mechanical prophylaxis of thromboembolic disease after total hip arthroplasty? | Excluded  Reason: Not RCT |
| 28. | Sharfman Z. et al, | 2016 | Balancing Thromboprophylaxis and Bleeding in Total Joint Arthroplasty: Impact of Eliminating Enoxaparin and Predonation and Implementing Pneumatic Compression and Tranexamic Acid | Excluded  Reason: Not RCT |
| 29. | Cao bing Y. et al, | 2010 | Rivaroxaban versus enoxaparin for thromboprophylaxis after total hip or knee arthroplasty: a meta-analysis of randomized controlled trials | Excluded  Reason: Not RCT |
| 30. | Trkulja V. et al, | 2010 | Rivaroxaban vs dabigatran for thromboprophylaxis after joint-replacement surgery: exploratory indirect comparison based on meta-analysis of pivotal clinical trials | Excluded  Reason: Not RCT |
| 31. | Ivanic M G. et al, | 2006 | Intermittent compression devices for swelling reduction and thrombosis prophylaxis--a pilot study after total hip replacement. Is the 2 hour daily minimum application sufficient? | Excluded  Reason: Not RCT |
| 32. | Jo Woo-Lam et al, | 2016 | Preventing Venous Thromboembolism with Use of Intermittent Pneumatic Compression after Total Hip Arthroplasty in Korean Patients | Excluded  Reason: Not RCT |
| 33. | Pour A. et al, | 2012 | Is venous foot pump effective in prevention of thromboembolic disease after joint arthroplasty: a meta-analysis | Excluded  Reason: Not RCT |
| 34. | Mont M. et al, | 2011 | AAOS clinical practice guideline: preventing venous thromboembolic disease in patients undergoing elective hip and knee arthroplasty | Excluded  Reason: Not RCT |
| 35. | Dohm M. et al, | 2011 | Micro-mobile foot compression device compared with pneumatic compression device | Excluded  Reason: Not RCT |
| 36. | Nagase Y. et al, | 2011 | Risk factors for pulmonary embolism and the effects of fondaparinux after total hip and knee arthroplasty: a retrospective observational study with use of a national database in Japan | Excluded  Reason: Not RCT |
| 37. | Sharrock N. et al, | 2008 | Potent anticoagulants are associated with a higher all-cause mortality rate after hip and knee arthroplasty | Excluded  Reason: Not RCT |
| 38. | Jun Liu et al, | 2024 | Effect of intermittent compression therapy on the prevention of deep venous thrombosis after hip arthroplasty: A systematic review and meta-analysis | Excluded  Reason: Not RCT |
| 39. | Wei Fengai et al, | 2021 | Prevention of venous thrombosis over the lower limbs after total hip arthroplasty by utilization of pneumatic therapy in addition to rivaroxaban | Excluded  Reason: Not RCT |
| 40. | Kurtoglu M. et al, | 2013 | Venous Thrombo-embolism prophylaxis: Intermittent pneumatic compression | Excluded  Reason: Not RCT |
| 41. | Hatano M. et al, | 2022 | Association Between Simultaneous Bilateral Total Hip Arthroplasty Without Any Anticoagulant or Antiplatelet Therapy and Deep Venous Thrombosis: A Cohort Study | Excluded  Reason: Not RCT |
| 42. | Lu Ning et al, | 2010 | Multimodal prophylaxis for venous thromboembolic disease after total hip and knee arthroplasty: current perspectives | Excluded  Reason: Not RCT |
| 43. | Falck-Ytter Y. et al, | 2012 | Prevention of VTE in Orthopedic Surgery Patients: Antithrombotic Therapy and Prevention of Thrombosis, 9th ed: American College of Chest Physicians Evidence-Based Clinical Practice Guidelines | Excluded  Reason: Not RCT |
| 44. | Berner J. et al, | 2021 | Alternative physical treatments for deep venous thrombosis prophylaxis in surgical patients: a systematic review | Excluded  Reason: Not RCT |
| 45. | Suna k. et al, | 2020 | Graduated compression stockings in the prevention of postoperative pulmonary embolism. A propensity-matched retrospective case-control study of 24 273 patients | Excluded  Reason: Not RCT |
| 46. | Garcez Leme L. et al, | 2012 | PROPHYLAXIS OF VENOUS THROMBOEMBOLISM IN ORTHOPAEDIC SURGERY | Excluded  Reason: Not RCT |
| 47. | Saleh J. et al, | 2017 | Deep Vein Thrombosis and Pulmonary Embolism Considerations in Orthopedic Surgery | Excluded  Reason: Not RCT |
| 48. | Comerota A. et al, | 2018 | Intermittent pneumatic compression is a cost-effective method of orthopedic postsurgical venous thromboembolism prophylaxis | Excluded  Reason: Not RCT |
| 49. | Connell S. et al, | 2016 | The Use of Intermittent Pneumatic Compression in Orthopedic and Neurosurgical Postoperative Patients | Excluded  Reason: Not RCT |
| 50. | Kwok M. et al, | 2013 | Stratified Meta-Analysis of Intermittent Pneumatic Compression of the Lower Limbs to Prevent Venous Thromboembolism in Hospitalized Patients | Excluded  Reason: Not RCT |
| 51. | Sadaghianloo N. et al, | 2016 | The efficacy of intermittent pneumatic compression in the prevention of lower extremity deep venous thrombosis | Excluded  Reason: Not RCT |
| 52. | Kim Young N. et al, |  | Effects of intermittent pneumatic compression devices interventions to prevent deep vein thrombosis in surgical patients: A systematic review and meta-analysis of randomized controlled trials | Excluded  Reason: Not RCT |
| 53. | Wang D. et al, | 2018 | Semiautomatic intermittent pneumatic compression device applied to deep vein thrombosis in major orthopedic surgery | Excluded  Reason: Not RCT |
| 54. | MacLellan D.et al, | 2007 | MECHANICAL COMPRESSION IN THE PROPHYLAXIS OF VENOUS THROMBOEMBOLISM | Excluded  Reason: Not RCT |
| 55. | Ryu S. et al, | 2024 | Characteristics and Effects of Intermittent Pneumatic Compression Devices Interventions to Prevent Venous Thromboembolism in Critical Care Patients: A Systematic Review of Randomized Controlled Trials | Excluded  Reason: Not RCT |
| 56. | BS Fan C. et al, | 2020 | Adjunctive Intermittent Pneumatic Compression in Hospitalized Patients Receiving Pharmacologic Prophylaxis for Venous Thromboprophylaxis: A Systematic Review and Meta-Analysis | Excluded  Reason: Not RCT |
| 57. | Braithwaite Irene | 2016 | Cohort feasibility study of an intermittent pneumatic compression device within a below-knee cast for the prevention of venous thromboembolism | Excluded  Reason: Not RCT |
| 58. | Amanatullah D. et al, | 2020 | Mechanical compression augments venous flow equal to intermittent pneumatic compression | Excluded  Reason: Not RCT |
| 59. | Zhang S. et al, | 2022 | Hemodynamic analysis of intermittent pneumatic compression combined with hyperthermia after total hip arthroplasty: an experiment on male rabbits | Excluded  Reason: Not RCT |
| 60. | Morris RJ. Et al, | 2010 | Intermittent Pneumatic Compression or Graduated Compression Stockings for Deep Vein Thrombosis Prophylaxis? | Excluded  Reason: Not RCT |
| 61. | Arabi Y. et al, | 2013 | Use of Intermittent Pneumatic Compression and Not Graduated Compression Stockings Is Associated With Lower Incident VTE in Critically Ill Patients: A Multiple Propensity Scores Adjusted Analysis | Excluded  Reason: Not RCT |
| 62. | Berliner J. et al, | 2018 | Venous Hemodynamics After Total Hip Arthroplasty: A Comparison Between Portable vs Stationary Pneumatic Compression Devices and the Effect of Body Position | Excluded  Reason: Not RCT |
| 63. | Lippi Giuseppe | 2011 | Prevention of Venous Thromboembolism: Focus on Mechanical Prophylaxis | Excluded  Reason: Not RCT |
| 64. | Anthony J. et al, | 2011 | Intermittent Pneumatic Compression for DVT Prophylaxis | Excluded  Reason: Not RCT |
| 65. | Epstein NE. et al, | 2005 | Intermittent Pneumatic Compression Stocking Prophylaxis Against Deep Venous Thrombosis in Anterior Cervical Spinal Surgery | Excluded  Reason: Not RCT |
| 66. | Kakkos K. et al, | 2020 | Interpretation of the PREVENT study findings on the adjunctive role of intermittent pneumatic compression to prevent venous thromboembolism | Excluded  Reason: Not RCT |
| 67. | Tang A. et al, | 2021 | Low-Dose Aspirin Thromboprophylaxis Is Safe and Effective in Patients Undergoing Total Hip Arthroplasty with or without Outpatient Pneumatic Compression Device | Excluded  Reason: Not RCT |
| 68. | Zareba P. et al, | 2014 | Meta-analysis of randomized trials comparing combined compression and anticoagulation with either modality alone for prevention of venous thromboembolism after surgery | Excluded  Reason: Not RCT |
| 69. | RN Ricky et al, | 2011 | Evidence based venous thromboprophylaxis in patients undergoing total hip replacement (THR), total knee replacement (TKR) and hip fracture surgery (HFS) | Excluded  Reason: Not RCT |
| 70. | Herring B. et al, | 2023 | A Systematic review of venous thromboembolism Mechanical prophylaxis during surgery. | Excluded  Reason: Not RCT |
| 71. | Yogendrakumar V. et al, | 2018 | Comparing pharmacological venous thromboembolism prophylaxis to intermittent pneumatic compression in acute intracerebral haemorrhage: protocol for a systematic review and network meta-analysis | Excluded  Reason: Not RCT |
| 72. | Caprini JA. Et al, | 2009 | Intermittent pneumatic compression and pharmacologic thrombosis prophylaxis | Excluded  Reason: Not RCT |
| 73. | O’Reilly RF. Et al, | 2005 | The prevalence of venous thromboembolism after hip and knee replacement surgery | Excluded  Reason: Not RCT |
| 74 | Junior DST. et al, | 2020 | Thromboprophylaxis Measures to Prevent Venous Thromboembolism in Hospitalized Patients | Excluded  Reason: Not RCT |
| 75. | Wong D. et al, | 2024 | Incidence of Venous Thromboembolism after Primary Total Hip Arthroplasty with Mechanical Prophylaxis in Hong Kong Chinese | Excluded  Reason: Not RCT |
| 76. | Morris R. et al, | 2006 | The influence of inflation rate on the hematologic and hemodynamic effects of intermittent pneumatic calf compression for deep vein thrombosis prophylaxis | Excluded  Reason: Not RCT |
| 77. | Yu X. et al, | 2021 | The deep vein thrombosis of lower limb after Total Hip arthroplasty: what should be care | Excluded  Reason: Not RCT |
| 78. | Pannucci CJ. et al, | 2016 | Benefits and Risks of Prophylaxis for Deep Venous Thrombosis and Pulmonary Embolus in Plastic Surgery | Excluded  Reason: Not RCT |
| 79. | Kakkos Sk. et al, | 2005 | Comparison of two intermittent pneumatic compression systems: A hemodynamic study | Excluded  Reason: Not RCT |
| 80. | Jr Pellegrini VD et al, | 2015 | Prophylaxis Against Venous Thromboembolism After Total Hip and Knee Arthroplasty | Excluded  Reason: Not RCT |
| 81. | Moheimani F. et al, | 2011 | Venous Thromboembolism: Classification, Risk Factors, Diagnosis, and Management | Excluded  Reason: Not RCT |
| 82. | Prestia BM. Et al, | 2020 | Are pneumatic compression devices as effective as anticoagulants for VTE prophylaxis after joint replacement surgery? | Excluded  Reason: Not RCT |
| 83. | Lewis S. et al, | 2019 | Venous Thromboembolism Prophylaxis Strategies for People Undergoing Elective Total Hip Replacement: A Systematic Review and Network Meta-Analysis | Excluded  Reason: Not RCT |
| 84. | Jiang T. et al, | 2022 | Thigh-length graduated compression stocking cannot increase blood velocity of the common femoral vein in patients awaiting total hip arthroplasty | Excluded  Reason: Not RCT |
| 85. | Kapoor A. et al, | 2017 | Comparative effectiveness of venous thromboembolism prophylaxis options for the patient undergoing total hip and knee replacement: a network meta‐analysis | Excluded  Reason: Not RCT |
| 86. | Gohal BM. et al, | 2017 | The Efficacy of Intermittent Pneumatic Compression Device as an Alternative Measure to Pharmacological Prophylaxis for Venous Thromboembolism in Postoperative and Post-Trauma Patients: An Integrative Review | Excluded  Reason: Not RCT |
| 87. | Muscatelli SR. et al, | 2021 | Time for an Update? A Look at Current Guidelines for Venous Thromboembolism Prophylaxis After Hip and Knee Arthroplasty and Hip Fracture | Excluded  Reason: Not RCT |
| 88. | Afshari A. et al, | 2018 | European guidelines on perioperative venous thromboembolism prophylaxis | Excluded  Reason: Not RCT |
| 89. | Xing K. et al, | 2008 | Has the incidence of deep vein thrombosis in patients undergoing total hip/knee arthroplasty changed over time? A systematic review of randomized controlled trials | Excluded  Reason: Not RCT |
| 90. | Kim K. et al, | 2013 | Thromboprophylaxis for Deep Vein Thrombosis and Pulmonary Embolism after Total Joint Arthroplasty in a Low Incidence Population. | Excluded  Reason: Not RCT |
| 91. | Epstein N. et al, | 2012 | A review of the risks and benefits of differing prophylaxis regimens for the treatment of deep venous thrombosis and pulmonary embolism in neurosurgery | Excluded  Reason: Not RCT |
| 92. | Migita K. et al, | 2014 | Venous thromboembolism after total joint arthroplasty: results from a Japanese multicenter cohort study | Excluded  Reason: Not RCT |
| 93. | Cayley Jr W. et al, | 2007 | Preventing Deep Vein thrombosis in hospital inpatients | Excluded  Reason: Not RCT |
| 94. | Rezaie A. et al, | 2018 | Venous thromboembolism prophylaxis after hip preservation surgery: a review and presentation of institutional experience | Excluded  Reason: Not RCT |
| 95. | Friedman RJ. et al, | 2010 | Practice Patterns in the Use of Venous Thromboembolism Prophylaxis After Total Joint Arthroplasty—Insights From the Multinational Global Orthopaedic Registry (GLORY) | Excluded  Reason: Not RCT |
| 96. | Raju KP. et al, | 2017 | Comparison between physiotherapeutic alone and combined physiotherapeutic and pharmacological measures in deep venous thrombosis prophylaxis in joint replacement and spinal surgeries | Excluded  Reason: Not RCT |
| 97. | Khatkar H. et al, | 2022 | Preventing Venous Thromboembolism after elective total hip arthroplasty surgery – are the current guidelines appropriate? Venous thromboembolism prophylaxis in elective total hip arthroplasty surgery | Excluded  Reason: Not RCT |
| 98. | Tanaka K. et al, | 2021 | Feasibility and Safety of a Novel Leg Exercise Apparatus for Venous Thromboembolism Prophylaxis after Total Joint Arthroplasty of the Lower Extremities—A Pilot Study | Excluded  Reason: Not RCT |
| 99. | Fisher W. et al, | 2011 | Impact of venous thromboembolism on clinical management and therapy after hip and knee arthroplasty | Excluded  Reason: Not RCT |
| 100. | Zhang Z. et al, | 2019 | Incidence and Risk Factors for Post-Thrombotic Syndrome in Patients With Deep Vein Thrombosis Following Total Knee and Hip Arthroplasty | Excluded  Reason: Not RCT |
| 101. | Almegren M. et al, | 2018 | Venous thromboembolism after total knee and hip Arthroplasty. | Excluded  Reason: Not RCT |
| 102. | Gu S. et al, | 2023 | Effect of different application duration of a venous foot pump on prevention of venous thromboembolism after hip and knee arthroplasty: a multicenter prospective clinical trial | Excluded  Reason: Not RCT |
| 103. | Samama CM. et al, | 2022 | Postoperative Venous Thromboembolism Prophylaxis: Changes in the Daily Clinical Practice, Modified Guidelines | Excluded  Reason: Not RCT |
| 104. | Bang SM. Et al, |  | Korean guidelines for the prevention of Venous thromboembolism | Excluded  Reason: Not RCT |
| 105. | Khan SA. et al, | 2020 | The incidence of venous thromboembolism in total joint replacement during COVID-19 pandemic | Excluded  Reason: Not RCT |
| 106. | Hou H. et al, | 2016 | Does intermittent pneumatic compression increase the risk of pulmonary embolism in deep venous thrombosis after joint surgery? | Excluded  Reason: Not RCT |
| 107. | Adam SS. Et al, | 213 | Clinical efficacy of risk-stratified prophylaxis with low-dose aspirin for the management of symptomatic venous thromboembolism after total hip arthroplasty | Excluded  Reason: Not RCT |
| 108. | Jamenson SS. Et al, | 2010 | The impact of national guidelines for the prophylaxis of venous thromboembolism on the complications of arthroplasty of the lower limb | Excluded  Reason: Not RCT |
| 109. | Zeng GJ. Et al, | 2020 | Incidence of deep vein thrombosis and pulmonary embolism in Asian patients after direct anterior total hip arthroplasty | Excluded  Reason: Not RCT |
| 110. | Forster R. et al, | 2016 | Anticoagulants (extended duration) for prevention of venous thromboembolism following total hip or knee replacement or hip fracture repair | Excluded  Reason: Not RCT |
| 111. | Uchida M. et al, | 2011 | Verification of the Effect of Techniques Used to Prevent Deep-vein Thrombosis | Excluded  Reason: Not RCT |
| 112. | Poultsides LA. et al, | 2012 | Meta-analysis of cause of death following total joint replacement using different thromboprophylaxis regimens | Excluded  Reason: Not RCT |
| 113. | Kordzadeh A. et al, | 2014 | Intermittent Pneumatic Compression in Treatment of Chronic Venous Leg Ulcers: A Case Report and Review of Literature | Excluded  Reason: Not RCT |
| 114. | Mahmoudi M. et al, | 2013 | The Cost-Effectiveness of Oral Direct Factor Xa Inhibitors Compared with Low-Molecular-Weight Heparin for the Prevention of Venous Thromboembolism Prophylaxis in Total Hip or Knee Replacement Surgery | Excluded  Reason: Not RCT |
| 115. | Gali J. et al, | 2017 | Deep Vein Thrombosis Prevention in Total Knee Arthroplasty. A Review | Excluded  Reason: Not RCT |
| 116. | Singh V. et al, | 2021 | Postoperative venous thromboembolism event increases risk of readmissions and reoperation following total joint arthroplasty: a propensity-matched cohort study | Excluded  Reason: Not RCT |
| 117. | Edward J.et al, | 2007 | Portable compression device and low-molecular-weight heparin compared with low-molecular-weight heparin for thromboprophylaxis after total joint arthroplasty | Excluded  Reason- not full text |
| 118. | Froimson M. et al, | 2009 | Venous Thromboembolic Disease Reduction With a Portable Pneumatic Compression Device | Excluded  Reason- not full text |
| 119. | Bern M. et al, | 2007 | Low-Dose Warfarin Coupled With Lower Leg Compression Is Effective Prophylaxis Against Thromboembolic Disease After Hip Arthroplasty | Excluded  Reason- not full text |
| 120. | Cooray R. et al, | 2015 | Prevention of deep vein thrombosis and pulmonary embolism | Excluded  Reason- not full text |
| 121. | Jinghui Y. et al, | 2020 | Study on the optimal time of using intermittent pneumatic compression after total hip arthroplasty: a randomized controlled trial | Excluded  Reason- not full text |
| 122. | Fujisawa M. et al | 2004 | Effect of calf-thigh intermittent pneumatic compression device after total hip arthroplasty: comparative analysis with plantar compression on the effectiveness of reducing thrombogenesis and leg swelling | Excluded  Reason- not full text |
| 123. | Tyagi Vineet et al, | 2017 | [The Role of Intraoperative Intermittent Pneumatic Compression Devices in Venous Thromboembolism Prophylaxis in Total Hip and Total Knee Arthroplasty](https://journals.healio.com/doi/full/10.3928/01477447-20171114-06) | Excluded  Reason- not full text |
| 124. | Andrews et. Al, | 2016 | Different Types of Intermittent Pneumatic Compression Devices for Preventing Venous Thromboembolism in Patients After Total Hip Replacement | Excluded  Reason- not full text |
| 125. | Pitto R.P. et al, | 2004 | Mechanical prophylaxis of deep-vein thrombosis after total hip replacement | Excluded  Reason- not full text |
| 126. | Jianwen FEI. Et al, | 2012 | Prevention and treatment of venous thromboembolism in elderly patients after joint replacement. | Excluded  Reason- not full text |
| 127. | Phillips L. et al, | 2004 | Review of Intermittent Pneumatic Compression: An Evidence-Based Therapeutic Modality for the Prevention of Avoidable Deep Vein Thrombosis | Excluded  Reason- not full text |
| 128. | Haas SB. et al, | 2008 | Venous thromboembolism Disease total hip and knee arthroplasty | Excluded  Reason- not full text |
| 129. | Pitto R. et al, | 2007 | Foot pumps without graduated compression stockings for prevention of deep-vein thrombosis in total joint replacement. | Excluded  Reason- not full text |
| 130. | Warwick D. et al, | 2011 | A Mobile Compression Device Compared With Low-Molecular-Weight Heparin for Prevention of Venous Thromboembolism in Total Hip Arthroplasty | Excluded  Reason- not full text |
| 131. | Della Valle AG. et al, | 2009 | Effect of Intermittent Pneumatic Compression on The Prevention of Postoperative Deep Venous Thrombosis During Laparoscopic Radical Resection Oof Rectal Cancer | Excluded  Reason- not full text |
| 132. | Sarmiento A. et al, | 2005 | Thromboembolic Disease Prophylaxis in Total Hip Arthroplasty | Excluded  Reason- not full text |
| 133. | Rawat A. et al, | 2008 | Primary Prophylaxis of Venous Thromboembolism in Surgical Patients | Excluded  Reason- not full text |
| 134. | Budhiparama N. et al, | 2014 | Venous Thromembolism Prophylaxis for Hip and Knee Arthroplasty: Changing trends | Excluded  Reason- not full text |
| 135. | Czyrny JJ. Et al, | 2010 | Electrical Foot stimulation: a potential new method of deep vein thrombosis Prophylaxis | Excluded  Reason- not full text |
| 136. | Broderick B. et al, | 2014 | Comparative lower limb hemodynamics using neuromuscular electrical stimulation (NMES) versus intermittent pneumatic compression (IPC) | Excluded  Reason- not full text |
| 137. | Schiff R. et al, | 2005 | Identifying Orthopedic Patients at High Risk for Venous Thromboembolism Despite Thromboprophylaxis | Excluded  Reason- not full text |
| 138. | Urbankova J. et al, | 2006 | **Intermittent pneumatic compression and deep vein thrombosis prevention in postoperative patients** | Excluded  Reason- not full text |
| 139. | Ishibe M. et al, | 2011 | Deep Venous Thrombosis after Mini-Posterior Total Hip Arthroplasty in Japanese Patients | Excluded  Reason- not full text |
| 140. | Salvati EA. et al, | 2005 | THE JOHN CHARNLEY AWARD: Heritable Thrombophilia and Development of Thromboembolic Disease after Total Hip Arthroplasty. | Excluded  Reason- not full text |
| 141. | Keeney J. et al, | 2006 | Efficacy of Combined Modality Prophylaxis Including Short-Duration Warfarin to Prevent Venous Thromboembolism After Total Hip Arthroplasty | Excluded  Reason- not full text |
| 142. | Sugano N. et al, | 2009 | Clinical Efficacy of Mechanical Thromboprophylaxis Without Anticoagulant Drugs for Elective Hip Surgery in an Asian Population | Excluded  Reason- not full text |
| 143. | Beksac B. et al, | 2007 | Symptomatic Thromboembolism after One-stage Bilateral THA with a Multimodal Prophylaxis Protocol. | Excluded  Reason- not full text |
| 144. | Kumazaki R. et al, | 2022 | Passive ankle motion and calf massage without anticoagulation therapy after total hip arthroplasty: A retrospective study | Excluded  Reason- not full text |
| 145. | Dohm M. et al, | 2011 | **Micro-mobile Foot Compression Device Compared with Pneumatic Compression Device** | Excluded  Reason- not full text |
| 146. | MO Z. et al, | 2016 | The preventive effect of rivaroxaban combine with intermittent pneumatic compression in patients after hip replacement | Excluded  Reason- not full text |
| 147. | Hass S. et al | 2006 | Prevention of major venous thromboembolism following total hip or knee replacement: a randomized comparison of low-molecular-weight heparin with unfractionated heparin (ECHOS Trial) | Excluded  Reason- not full text |
| 148. | Mont M. et al, | 2011 | AAOS clinical practice guideline: preventing venous thromboembolic disease in patients undergoing elective hip and knee arthroplasty | Excluded  Reason- not full text |
| 149. | Rinehart D.et al, | 2021 | The Current Status of Venous Thromboembolism Prophylaxis Following Total Joint Arthroplasty | Excluded  Reason- not full text |
| 150. | Long JB. Et al, | 2009 | Venous Thromboembolism: Pharmacological and Nonpharmacological Interventions | Excluded  Reason- not full text |
| 151. | Swanson E. et al, | 2015 | Do Sequential Compression Devices Really Reduce the Risk of Venous Thromboembolism in Plastic Surgery Patients? | Excluded  Reason- not full text |
| 152. | Bern M. et al, | 2007 | Low-Dose Warfarin Coupled With Lower Leg Compression Is Effective Prophylaxis Against Thromboembolic Disease After Hip Arthroplasty. | Excluded  Reason- not full text |
| 153. | Tsuda K. et al, | 2020 | Asymptomatic Deep Venous Thrombosis After Elective Hip Surgery Could Be Allowed to Remain in Place Without Thromboprophylaxis After a Minimum 2-Year Follow-Up | Excluded  Reason- not full text |
| 154. | Kim Y. et al, | 2015 | Prevalence of Deep Vein Thrombosis and Pulmonary Embolism Treated with Mechanical Compression Device after Total Hip Arthroplasty | Excluded  Reason- not full text |
| 155. | Levitan B. et al, | 2014 | Benefit-risk assessment of rivaroxaban versus enoxaparin for the prevention of venous thromboembolism after total hip or knee arthroplasty | Excluded Reasons = IPC not as intervention |
| 156. | Cao Bing Y. et al, | 2010 | Rivaroxaban versus enoxaparin for thromboprophylaxis after total hip or knee arthroplasty: a meta-analysis of randomized controlled trials | Excluded Reasons = IPC not as intervention |
| 157. | Kwong L. rt al, | 2011 | Therapeutic potential of rivaroxaban in the prevention of venous thromboembolism following hip and knee replacement surgery: a review of clinical trial data | Excluded Reasons = IPC not as intervention |
| 158. | Gomez-Outes A. et al, | 2012 | Dabigatran, rivaroxaban, or apixaban versus enoxaparin for thromboprophylaxis after total hip or knee replacement: systematic review, meta-analysis, and indirect treatment comparisons | Excluded Reasons = IPC not as intervention |
| 159. | Loke K Y. et al, | 2011 | Dabigatran and rivaroxaban for prevention of venous thromboembolism--systematic review and adjusted indirect comparison | Excluded Reasons = IPC not as intervention |
| 160. | Cohen A., et al, | 2012 | The efficacy and safety of pharmacological prophylaxis of venous thromboembolism following elective knee or hip replacement: systematic review and network meta-analysis | Excluded Reasons = IPC not as intervention |
| 161. | Nakase J. et al, | 2009 | Heparin versus danaproid for prevention of venous thromboembolism after hip surgery | Excluded Reasons = IPC not as intervention |
| 162. | Farey J. et al, | 2021 | Aspirin versus enoxaparin for the initial prevention of venous thromboembolism following elective arthroplasty of the hip or knee: A systematic review and meta-analysis | Excluded Reasons = IPC not as intervention |
| 163. | Deirmengian G. et al, | 2016 | Aspirin Can Be Used as Prophylaxis for Prevention of Venous Thromboembolism After Revision Hip and Knee Arthroplasty | Excluded Reasons = IPC not as intervention |
| 164. | Imai N. et al, | 2017 | Manual calf massage and passive ankle motion reduce the incidence of deep vein thromboembolism after total hip arthroplasty | Excluded Reasons = IPC not as intervention |
| 167. | Fuji T. et al, | 2014 | Safety and Efficacy of Edoxaban, an Oral Factor Xa Inhibitor, for Thromboprophylaxis After Total Hip Arthroplasty in Japan and Taiwa | Excluded Reasons = IPC not as intervention |
| 168. | Lee GW. Et al, | 2017 | New strategy of closed suction drainage after primary total hip arthroplasty | Excluded Reasons = IPC not as intervention |
| 169. | Gelfer Y.et al | 2006 | Deep Vein Thrombosis (DVT) Prevention in Total Hip Arthroplasty: continuous Enhanced Circulation Therapy (CECT) Versus Low Molecular Weight Heparin (LMWH) | Excluded Reasons = IPC not as intervention |
| 170. | Yilmaz M. et al, | 2023 | Aspirin is an Effective Prophylaxis for Venous Thromboembolism After Revision Hip and Knee Arthroplasty | Excluded Reasons = IPC not as intervention |
| 171. | Manfredi VM. Et al, | 2021 | Effectiveness of deep vein thrombosis prevention in Total Hip Arthroplasty. | Excluded Reasons = IPC not as intervention |
| 172. | Woon C. et al, | 2018 | Aspirin Alone Is Not Enough to Prevent Deep Vein Thrombosis After Total Joint Arthroplsty. | Excluded Reasons = IPC not as intervention |
| 173. | Deirmengian GK. Et al, | 2016 | Aspirin Can Be Used as Prophylaxis for Prevention of Venous Thromboembolism After Revision Hip and Knee Arthroplasty | Excluded Reasons = IPC not as intervention |
| 174. | Yukizawa Y. et al, | 2011 | Association between venous thromboembolism and plasma levels of both soluble fibrin and plasminogen-activator inhibitor 1 in 170 patients undergoing total hip arthroplasty | Excluded Reasons = IPC not as intervention |
| 175. | Imai N. et al, | 2017 | Manual calf massage and passive ankle motion reduce the incidence of deep vein thromboembolism after total hip arthroplasty | Excluded Reasons = IPC not as intervention |
| 176. | Kessler P. et al, | 2006 | Prevention of venous thromboembolism in orthopedics | Excluded Reasons = IPC not as intervention |
| 177. | Yamaguchi T. et al, | 2010 | Incidence and time course of asymptomatic deep vein thrombosis with fondaparinux in patients undergoing total joint arthroplasty | Excluded Reasons = IPC not as intervention |
| 178. | Lassen M. et al, | 2004 | Postoperative fondaparinux versus preoperative enoxaparin for prevention of venous thromboembolism in elective hip-replacement surgery: a randomised double-blind comparison | Excluded Reasons = IPC not as intervention |
| 179. | Eriksson B. et al, | 2006 | Oral, direct Factor Xa inhibition with BAY 59-7939 for the prevention of venous thromboembolism after total hip replacement | Excluded Reasons = IPC not as intervention |
| 180. | CRISTAL EASG | 2024 | Cost-effectiveness of enoxaparin versus aspirin in the prevention of venous thromboembolism after total hip or knee arthroplasty: an analysis from the CRISTAL cluster-randomized trial | Excluded Reasons = IPC not as intervention |
| 181. | Eriksoon BI. Et al, | 2007 | Dabigatran etexilate versus enoxaparin for prevention of venous thromboembolism after total hip replacement: a randomised, double-blind, non-inferiority trial | Excluded Reasons = IPC not as intervention |
| 182. | Azboy I. et al, | 2017 | Aspirin and the prevention of venous thromboembolism following total joint arthroplasty | Excluded Reasons = IPC not as intervention |
| 183. | Kakkar A. et al, | 2008 | A prediction nomogram for deep venous thrombosis risk in patients undergoing primary total hip and knee arthroplasty: a retrospective study | Excluded Reasons = IPC not as intervention |
| 184. | Trivedi NN. et al, | 2019 | Venous Thromboembolism Chemoprophylaxis in Total Hip and Knee Arthroplasty | Excluded Reasons = IPC not as intervention |
| 185. | Maradei-Pereiara J. et al | 2022 | Thromboprophylaxis with unilateral pneumatic device led to less edema and blood loss compared to enoxaparin after knee arthroplasty: randomized trial | Excluded  Reason – not THA patient |
| 186. | Dietz M. et al, | 2022 | Randomized Trial of Postoperative Venous Thromboembolism Prophylactic Compliance: Aspirin and Mobile Compression Pumps | Excluded  Reason – not THA patient |
| 187. | Snyder M. et al, | 2017 | Efficacy in Deep Vein Thrombosis Prevention With Extended Mechanical Compression Device Therapy and Prophylactic Aspirin Following Total Knee Arthroplasty: A Randomized Control Trial | Excluded  Reason – not THA patient |
| 188. | Kahn S. et al, | 2012 | Prevention of VTE in Nonsurgical Patients: Antithrombotic Therapy and Prevention of Thrombosis, 9th ed: American College of Chest Physicians Evidence-Based Clinical Practice Guidelines | Excluded  Reason – not THA patient |
| 189. | Morris J. et al, | 2012 | [Intermittent Pneumatic Compression for Venous Thromboembolism Prophylaxis in Total Knee Arthroplasty](https://journals.healio.com/doi/full/10.3928/01477447-20121120-15) | Excluded  Reason – not THA patient |
| 190. | Liu P. et al, | 2017 | Intermittent pneumatic compression devices combined with anticoagulants for prevention of symptomatic deep vein thrombosis after total knee arthroplasty: a pilot study | Excluded  Reason – not THA patient |
| 191. | Wang S. et al, | 2021 | Prevention of deep venous thrombosis in patients undergoing knee arthroplasty by intermittent pneumatic compression apparatus | Excluded  Reason – not THA patient |
| 192. | Lobastov K. et al, | 2021 | Intermittent Pneumatic Compression in Addition to Standard Prophylaxis of Postoperative Venous Thromboembolism in Extremely High-risk Patients (IPC SUPER): A Randomized Controlled Trial | Excluded  Reason – not THA patient |
| 193. | Domeij-Arverud E. et al, | 2015 | Intermittent pneumatic compression reduces the risk of deep vein thrombosis during post-operative lower limb immobilisation | Excluded  Reason – not THA patient |
| 194. | M. Arabi Y. et al, | 2019 | Adjunctive Intermittent Pneumatic Compression for Venous Thromboprophylaxis | Excluded  Reason – not THA patient |
| 195. | Greenall R. et al, | 2019 | Intermittent pneumatic compression for venous thromboembolism prevention: a systematic review on factors affecting adherence | Excluded  Reason – not THA patient |
| 196. | Turpie A.G.G et al, | 2007 | Fondaparinux combined with intermittent pneumatic compression vs. intermittent pneumatic compression alone for prevention of venous thromboembolism after abdominal surgery: a randomized, double‐blind comparison | Excluded  Reason – not THA patient |
| 197. | Kim K. et al, | 2019 | Pneumatic compression device does not show effective thromboprophylaxis following total knee arthroplasty in a low incidence population | Excluded  Reason – not THA patient |
| 198. | Nagahiro I. et al, | 2004 | Intermittent Pneumatic Compression is Effective in Preventing Symptomatic Pulmonary Embolism After Thoracic Surgery. | Excluded  Reason – not THA patient |
| 199. | Park Sin. et al, | 2016 | Incidences of Deep Vein Thrombosis and Pulmonary Embolism after Total Knee Arthroplasty Using a Mechanical Compression Device with and without Low-Molecular-Weight Heparin | Excluded  Reason – not THA patient |
| 200. | Windisch C. et al, | 2010 | Pneumatic Compression with foot pumps facilitates early postoperative mobilization in total knee arthroplasty. | Excluded  Reason – not THA patient |
| 201. | AI-Dorzi H. et al, | 2022 | The effect of intermittent pneumatic compression on deep-vein thrombosis and ventilation-free days in critically ill patients with heart failure | Excluded  Reason – not THA patient |
| 202. | Zhou X. et al, | 2020 | Elastic stockings plus enoxaparin and intermittent pneumatic compression in preventing postoperative deep venous thrombosis in patients with ovarian cancer | Excluded  Reason – not THA patient |
| 203. | Lachiewicz P.F. et al, | 2004 | Two mechanical devices for prophylaxis of thromboembolism after total knee arthroplasty | Excluded  Reason – not THA patient |
| 204. | Yogenderkumar V. et al, | 2018 | Comparing pharmacological venous thromboembolism prophylaxis to intermittent pneumatic compression in acute intracerebral haemorrhage: protocol for a systematic review and network meta-analysis | Excluded  Reason – not THA patient |
| 205. | Shin Won et al, | 2017 | Recent Updates of the Diagnosis and Prevention of Venous Thromboembolism in Patients with a Hip Fracture | Excluded  Reason – not THA patient |
| 206. | Chin PL. et al, | 2009 | Thromboembolism Prophylaxis for Total Knee Arthroplasty: A randomized controlled trail | Excluded  Reason – not THA patient |
| 207. | Giannoni MF. Et al, | 2006 | Total knee replacement: prevention of deep-vein thrombosis using pharmacological (low-molecular-weight heparin) and mechanical (intermittent foot sole pump system) combined prophylaxis | Excluded  Reason – not THA patient |
| 208. | Choi J. et al, | 2014 | Comparison of Simultaneous and Alternate Bilateral Pneumatic Compression in Hemodynamic Effects and Thromboprophylaxis After Total Knee Arthroplasty | Excluded  Reason – not THA patient |
| 209. | Nam J. et al, | 2017 | Does preoperative mechanical prophylaxis have additional effectiveness in preventing postoperative venous thromboembolism in elderly patients with hip fracture?—Retrospective case-control study | Excluded  Reason – not THA patient |
| 210 | Hou D. et al, | 2017 | Efficacy and safety of low molecular weight heparin treatment, intermittent pneumatic compression therapy passive ankle exercise and nursing care in preventing deep venous thrombosis of the lower extremity following varicose vein surgery. | Excluded  Reason – not THA patient |
| 211. | Zhong X. et al, | 2017 | Effect of Intermittent Pneumatic Compression on The Prevention of Postoperative Deep Venous Thrombosis During Laparoscopic Radical Resection Oof Rectal Cancer | Excluded  Reason – not THA patient |
| 212. | Moslehi M. et al, | 2015 | The Comparison of Incidence Deep Vein Thrombosis for Three Mechanical Methods of Graduated Compression Stockings (GCS),Intermittent Pneumatic Compression (IPC) and Elastic Bandage in Intensive Care Units | Excluded  Reason – not THA patient |
| 213. | Jiang L. et al, | 2017 | Stacked Modalities' Thromboprophylactic Therapy for Patients Undergoing Total Knee Replacement Surgery | Excluded  Reason – not THA patient |
| 214. | Ditez MJ. Et al, | 2022 | Randomized Trial of Postoperative Venous Thromboembolism Prophylactic Compliance: Aspirin and Mobile Compression Pumps | Excluded  Reason – not THA patient |
| 215. | Zhi-wen FAN et al, | 2021 | Clinical observation on intermittent pneumatic compression and low-molecular-weight heparin in the prevention of lower extremity deep venous thrombosis in medical ICU patients with high risk | Excluded  Reason – not THA patient |
| 216. | Fuji T. et al, | 2010 | A dose‐ranging study evaluating the oral factor Xa inhibitor edoxaban for the prevention of venous thromboembolism in patients undergoing total knee arthroplasty. | Excluded  Reason – not THA patient |
| 217. | Ono K. et al, | 2020 | Risk of deep venous thrombosis after total knee arthroplasty in patients with haemophilia A | Excluded  Reason – not THA patient |
| 218. | Sakai T. et al, | 2016 | Effects of a Foot Pump on the Incidence of Deep Vein Thrombosis After Total Knee Arthroplasty in Patients Given Edoxaban | Excluded  Reason – not THA patient |
| 219. | Jiang Y. et al, | 2014 | **Aspirin combined with mechanical measures to prevent venous thromboembolism after total knee arthroplasty: a randomized controlled trial** | Excluded  Reason – not THA patient |
| 220. | Tateiwa T. et al, | 2021 | Does intraoperative mechanical prophylaxis prevent venous thromboembolism in total knee arthroplasty? – effectiveness of passive-assisted ankle motion in surgical/non-surgical side | Excluded  Reason – not THA patient |
| 221. | Loh JLM. et al, | 2019 | Chemoprophylaxis in addition to mechanical prophylaxis after total knee arthroplasty surgery does not reduce the incidence of venous thromboembolism | Excluded  Reason – not THA patient |
| 222. | Stevens S. et al, | 2013 | Intermittent pneumatic compression in patients with stroke | Excluded  Reason – not THA patient |
| 223. | Kurtoglu M. et al, | 2005 | Intermittent pneumatic compression in the prevention of venous thromboembolism in high-risk trauma and surgical ICU patients | Excluded  Reason – not THA patient |
| 224. | Domeij-Arverud E. et al, | 2013 | Can foot compression under a plaster cast prevent deep-vein thrombosis during lower limb immobilisation? | Excluded  Reason – not THA patient |
| 225. | Li C. et al, | 2024 | The Effect of Intermittent Pneumatic Compression Device Combined with Low-Molecular-Weight Heparin on the Prevention of Deep Vein Thrombosis in Elderly Patients after Femoral Neck Fracture Surgery | Excluded  Reason – not THA patient |
| 226. | Jian-wei L. et al, | 2017 | Prevention of the perioperative deep venous thrombosis in hip Arthroplasty. | Excluded Reason - Preoperative population only |
| 227. | Zhou X. et al, | 2023 | Application Areas of Intermittent Pneumatic Compression in the Prevention of Deep Vein Thrombosis During Dixon Surgery: A Randomized, Controlled Trial | Excluded Reason - Preoperative population only |
| 228. | Chohan A. et al, | 2020 | Examination of a new mobile intermittent pneumatic compression device in healthy adults | Excluded Reason - Preoperative population only |
| 229. | Jenny JY. Et al, | 2018 | European guidelines on perioperative venous thromboembolism prophylaxis | Excluded Reason - Preoperative population only |
| 230. | Ellis M. et al, | 2004 | Perioperative venous thromboembolism prophylaxis in Israel: a survey of academic surgical departments | Excluded Reason - Preoperative population only |
| 231. | Yao Y. et al, | 2024 | An analysis of the prevalence and risk factors of deep vein thrombosis in non-fracture patients awaiting total hip arthroplasty: a retrospective study of 1244 cases | Excluded Reason - Preoperative population only |
| 232. | Citak M. et al, | 2015 | Are patients with preoperative air travel at higher risk for venous thromboembolism following primary total hip and knee arthroplasty? | Excluded Reason - Preoperative population only |
| 233. | Sidhu V. et al, | 2022 | Effect of Aspirin vs Enoxaparin on Symptomatic Venous Thromboembolism in Patients Undergoing Hip or Knee Arthroplasty: The CRISTAL Randomized Trial | Excluded Reason - Preoperative population only |
| 234. | Seward M. et al, | 2022 | Obesity, preoperative weight loss, and telemedicine before total joint arthroplasty: a review | Excluded Reason - Preoperative population only |
| 235. | Sochart S. et al, | 2023 | The use of pre-operative Inferior Vena Cava filters for thromboprophylaxis in ultra-high-risk patients undergoing total hip and knee arthroplasty: a systematic review and narrative analysis | Excluded Reason - Preoperative population only |
| 236. | Stowers M. et al, | 2014 | Review article: Perioperative care in enhanced recovery for total hip and knee arthroplasty | Excluded Reason - Preoperative population only |
| 237. | Plenge U. et al, | 2018 | Optimising perioperative care for hip and knee arthroplasty in South Africa: a Delphi consensus study | Excluded Reason - Preoperative population only |
| 238. | Larsen K. et al, | 2009 | Cost-effectiveness of accelerated perioperative care and rehabilitation after total hip and knee arthroplasty | Excluded Reason - Preoperative population only |
| 239. | Berend K. et al, | 2004 | Rapid recovery protocol for peri-operative care of total hip and total knee arthroplasty patients. | Excluded Reason - Preoperative population only |
| 240. | Wasko MK. Et al, | 2018 | D-dimer Levels are not a Good Marker for Venous Thromboembolism in Patients Before and After Total Hip Replacement. | Excluded Reason - Preoperative population only |
| 241 | Magill P. et al, | 2021 | Oral tranexamic acid for an additional 24 hours postoperatively versus a single preoperative intravenous dose for reducing blood loss in total hip arthroplasty | Excluded Reason - Preoperative population only |
| 242. | Cheng T. et al, | 2023 | Chronic Obstructive Pulmonary Disease is Associated With Serious Infection and Venous Thromboembolism in Patients Undergoing Hip or Knee Arthroplasties: A Meta-Analysis of Observational Studies | Excluded Reason - Preoperative population only |
| 243. | Park J S. et al, | 2018 | High energy injury is a risk factor for preoperative venous thromboembolism in the patients with hip fractures: A prospective observational study | Excluded Reason - Preoperative population only |
| 244. | Shin W. et al, | 2016 | Preoperative Prevalence of and Risk Factors for Venous Thromboembolism in Patients with a Hip Fracture: An Indirect Multidetector CT Venography Study | Excluded Reason - Preoperative population only |
| 245. | Petersen PB. Et al, | 2018 | Safety of In-Hospital Only Thromboprophylaxis after Fast-Track Total Hip and Knee Arthroplasty: A Prospective Follow-Up Study in 17,582 Procedures | Excluded Reason - Preoperative population only |
| 246. | Lou X. et al, | 2010 | Effect of different analgesia combined with low molecular heparin on hemorheology and coagulation in patients undergoing total hip replacement | Excluded Reason - Preoperative population only |
| 247. | Green L. et al, | 2010 | The impact of elective knee/hip replacement surgery and thromboprophylaxis with rivaroxaban or dalteparin on thrombin generation | Excluded Reason - Preoperative population only |
| 248. | Gionis M. et al, | 2013 | The study of the thrombin generation mechanism and the effect of low molecular weight heparin as thromboprophylaxis in patients undergoing total knee and hip replacement | Excluded Reason - Preoperative population only |
| 249 | Nam D. et al, | 2015 | Thromboembolism Prophylaxis in Hip Arthroplasty: Routine and High Risk Patients | Excluded Reason- Inappropriate Outcome Measures used. |
| 250. | Leegwater N. et al, | 2012 | Cryocompression therapy after elective arthroplasty of the hip | Excluded Reason- Inappropriate Outcome Measures used. |
| 251. | Leegwater N. et al, | 2016 | The efficacy of continuous-flow cryo and cyclic compression therapy after hip fracture surgery on postoperative pain: design of a prospective, open-label, parallel, multicenter, randomized controlled, clinical trial | Excluded Reason- Inappropriate Outcome Measures used. |
| 252. | Yao M. et al, | 2024 | **Comparison of intermittent pneumatic compression pump as adjunct to decongestive lymphatic therapy against decongestive therapy alone for upper limb lymphedema after breast cancer surgery: a systematic review and meta-analysis** | Excluded Reason- Inappropriate Outcome Measures used. |
| 253. | Yeroushalmi D. et al, | 2022 | Patient Satisfaction and Risk of Falls with the Use of Intermittent Pneumatic Compression Devices Following Total Joint Arthroplasty | Excluded Reason- Inappropriate Outcome Measures used. |
| 254. | McAsey J C. et al, | 2014 | Patient Satisfaction With Mobile Compression Devices Following Total hip Arthroplasty | Excluded Reason- Inappropriate Outcome Measures used. |
| 255. | Dorr LD et al, | 2007 | Multimodal Thromboprophylaxis for Total Hip and Knee Arthroplasty Based on Risk Assessment | Excluded Reason- Inappropriate Outcome Measures used. |
| 256. | Xu Z. et al, | 2013 | Postoperative Plasma D-Dimer Value for Predicting Deep Venous Thrombosis following Hip Arthroplasty with Nadroparin Prophylaxis | Excluded Reason- Inappropriate Outcome Measures used. |
| 257. | Lee W. et al, | 2018 | Investigation of Blood Flow During Intermittent Pneumatic Compression and Proposal of a New Compression Protocol | Excluded Reason- Inappropriate Outcome Measures used. |
| 258. | Williams KJ, et al, | 2014 | Haemodynamic changes with the use of neuromuscular electrical stimulation compared to intermittent pneumatic compression | Excluded Reason- Inappropriate Outcome Measures used. |
| 259. | Gardiner D.A. et al, | 2015 | Improving rates of Intermittent Pneumatic Compression therapy Utilization | Excluded Reason- Inappropriate Outcome Measures used. |
| 260. | Kavros S. et al, | 2008 | Improving limb salvage in critical ischemia with intermittent pneumatic compression: A controlled study with 18-month follow-up | Excluded Reason- Inappropriate Outcome Measures used. |
| 261. | Lin Z. et al, | 2023 | A prediction nomogram for deep venous thrombosis risk in patients undergoing primary total hip and knee arthroplasty: a retrospective study | Excluded Reason- Inappropriate Outcome Measures used. |
| 262. | Mihara M. et al, | 2020 | Clinical efficacy of risk-stratified prophylaxis with low-dose aspirin for the management of symptomatic venous thromboembolism after total hip arthroplasty | Excluded Reason- Inappropriate Outcome Measures used. |
| 263. | Colwell C. et al, | 2012 | Cost-Effectiveness of Venous Thromboembolism Prophylaxis With a New Mobile Device After Total Hip Arthroplasty | Excluded Reason- Inappropriate Outcome Measures used. |
| 264. | Ringwald J. et al, | 2008 | Genetic Polymorphisms in Venous Thrombosis and Pulmonary Embolism After Total Hip Arthroplasty: A Pilot Study | Excluded Reason- Inappropriate Outcome Measures used. |
