# Supplementary material for: Intermittent pneumatic compression therapy as a preventive measure for venous thromboembolism after total hip arthroplasty: A systematic review
Source: PLoS One. 2025 Jun 4;20(6):e0318954. doi: 10.1371/journal.pone.0318954 (PMC12136298; doi:10.1371/journal.pone.0318954)
Supplement: Attachment — Submitted filename: Review 26.pdf [file pone.0318954.s003.pdf]

Title: Intermittent pneumatic compression therapy as a preventive measure for venous thromboembolism after total hip arthroplasty: A systematic review

Journal: PLOS ONE

Reviewer: Seyed Amirhossein Mazhari

Reviewer's report:

The manuscript offers a comprehensive systematic review on the efficacy of intermittent pneumatic compression (IPC) as a prophylactic measure against VTE after total hip arthroplasty. The authors have followed PRISMA guidelines for their systematic search and analysis of RCTs, evaluating IPC alone or in combination with pharmacological agents.

The manuscript is well-structured and well-analyzed and provides valuable results. The tables and figures are appropriately depicted and Cleary presented. The Discussion section need to be more thorough and detailed. Study limitation are mentioned in a separate section in detail, adding additional strength to the article.

At the end I have some questions about this article.

The specific questions and recommendations are as follows:

1:
Conclusion parts in both the abstract and the main text needs to be rewritten and lengthened. Consider adding more supporting evidence.

2:
The discussion should be more comprehensive. Furthermore, The introduction presents an appropriate outline but it is better to be lengthened. Adding additional references is recommended in both introduction and discussion section.

3:
It is recommended to add a new section at the end of the article after conclusion section, describing any abbreviation which is being used throughout the article.
